# The role of NOP58 in prostate cancer progression through SUMOylation regulation and drug response

**DOI:** 10.3389/fphar.2024.1476025

**Published:** 2024-10-18

**Authors:** Wei Guo, Shi Zong, Tao Liu, Yi Chao, Kaichen Wang

**Affiliations:** Department of Urinary Surgery, The Third Bethune Hospital of Jilin University, Changchun, Jilin, China

**Keywords:** prostate cancer, SUMOylation, NOP58, bioinformatics, prognosis, single-cell analysis, post-translational modifications

## Abstract

**Background:**

Prostate cancer is one of the leading causes of cancer-related deaths in men. Its molecular pathogenesis is closely linked to various genetic and epigenetic alterations, including posttranslational modifications like SUMOylation. Identifying biomarkers that predict outcomes and specific therapeutic targets depends on a comprehensive understanding of these processes. With growing interest in SUMOylation as a mechanism affecting prostate cancer-related genes, this study aimed to investigate the central role of SUMOylation in prostate cancer prognostics, focusing on the significance of NOP58.

**Methods:**

We conducted a comprehensive bioinformatics analysis, integrating differential expression analysis, survival analysis, gene set enrichment analysis (GSEA), and single-cell transcriptomic analyses using data from The Cancer Genome Atlas (TCGA). Key genes were identified through intersections of Venn diagrams, Boralta algorithm signatures, and machine learning models. These signaling mechanisms were validated through experimental studies, including immunohistochemical staining and gene ontology analyses.

**Results:**

The dual-gene molecular subtype analysis with SUMO1, SUMO2, and XPO1 genes revealed significant differences in survival outcomes across molecular subtypes, further emphasizing the potential impact of NOP58 on SUMOylation, a key post-translational modification, in prostate cancer. NOP58 overexpression was strongly associated with shorter overall survival (OS), progression-free interval (PFI), and disease-specific death in prostate cancer patients. Immunohistochemical analysis confirmed that NOP58 was significantly overexpressed in prostate cancer tissues compared to normal tissues. ROC curve analysis demonstrated that NOP58 could distinguish prostate cancer from control samples with high diagnostic accuracy. Gene Ontology analysis, along with GSVA and GSEA, suggested that NOP58 may be involved in cell cycle regulation and DNA repair pathways. Moreover, NOP58 knockdown led to increased BCL2 expression and decreased Ki67 levels, promoting apoptosis and inhibiting cell proliferation. Colony formation assays further showed that NOP58 knockdown inhibited, while its overexpression promoted, colony formation, highlighting the critical role of NOP58 in prostate cancer cell growth and survival. Additionally, NOP58 was linked to drug responses, including Methotrexate, Rapamycin, Sorafenib, and Vorinostat.

**Conclusion:**

NOP58 is a key regulator of prostate cancer progression through its mediation of the SUMOylation pathway. Its expression level serves as a reliable prognostic biomarker and an actionable therapeutic target, advancing precision medicine for prostate cancer. Targeting NOP58 may enhance therapeutic efficacy and improve outcomes in oncology.

## Background

Prostate cancer (PCa) is one of the most prevalent male malignancies and its morbidity and mortality are high worldwide (Enikeeva et al., 2024). Although there has been significant progress in the diagnosis and treatment of prostate cancer over recent years, its molecular nature is complex and characterized by multiple interacting biological mechanisms which require comprehensive investigation (Nevedomskaya and Haendler, 2022; Wasim et al., 2022). Prostate cancer is a typical malignant carcinoma of the prostate, which has its own unique genes, and from this background many molecular mechanisms related to its onset have been clarified including genetic mutations, epigenetic modification, post-translational modifications, etc., in which SUMOylation is acrucial post-translational modification with wide biological functions (Samaržija, 2021; Sun et al., 2023).

SUMOylation (Small Ubiquitin-like Modifier) is a process in which SUMO proteins attach to target proteins post-translationally (Eifler and Vertegaal, 2015; Raju, 2019). This process is an important feature of numerous cellular biology processes such as transcriptional regulation, DNA repair, signal transduction and cell cycle control mechanisms (Soutourina and Werner, 2014; Tunçer and Kavak, 2020). SUMOylation is a reversible and dynamic posttranslational modification that modulates the stability, subcellular localization, and interaction networks of target proteins to regulate cell function and fate (Sahin et al., 2022; Huang et al., 2024). In recent years, a growing number of researches have demonstrated that SUMOylation is associated with the initiation and progression of human cancer (Du et al., 2021; Qin et al., 2021).

Although much of the cancer research has already recognized the role of SUMOylation, its distinct mechanisms and actions in prostate cancer remain largely unknown (Vlachostergios and Papandreou, 2012; Wang and Yu, 2021). The occurrence and progression of prostate cancer are mediated by multiple molecular and cellular pathways, and SUMOylation may be involved in the development of PCa by regulating critical proteins in these pathways (Wang and Yu, 2021; Ballar Kirmizibayrak et al., 2020). The occurrence and progression of prostate cancer are mediated by multiple molecular and cellular pathways, and SUMOylation may be involved in the development of PCa by regulating critical proteins in these pathways (Sun et al., 2023; Wu et al., 2020).

The specific objectives of this study were to discover the key genes associated with SUMOylation activity that could predict the prognosis of patients with prostate cancer (Sun et al., 2023; Liu et al., 2020). Bioinformatics methods, such as differential expression analysis, survival analysis, gene set enrichment analysis (GSEA), single-cell transcriptomics will be used to show how these genes are involved throughout the progression of prostate cancer (Khan et al., 2022; He et al., 2019). In the present study, we showed that NOP58 is a critical regulator in th SUMOylation pathway by comprehensive analysis. The diagnostic and therapeutic biomarker value of NOP58 was extended in this study by immunohistochemistry, gene ontology and pathway analysis.

Utilizing bioinformatics, the present study was conducted to identify and analyze the prognostic-related modification of SUMO ubiquitination in genes asso-ciated with prostate cancer (Sun et al., 2023; Wang and Yu, 2021). Utilizing bioinformatics, the present study was conducted to identify and analyze the prognostic-related modification of SUMO ubiquitination in genes asso-ciated with prostate cancer (Liu et al., 2022; Boldrini et al., 2021). Survival prognosis analysis of OS/PFI/DSS were then conducted; these results indicated that several genes might have a prognostic value in prostate cancer survival outcomes (Clayman et al., 2020; Reyes et al., 2021). The VennDiagram.R package was utilized to determine the intersection of OS-PFI-DSS related genes (Zhou et al., 2024; Chen et al., 2022). These intersecting prognostic genes were then subjected to binary Boruta analysis to pinpoint key genes closely linked to prostate cancer (Liu et al., 2020; He et al., 2019). In addition, a suite of ten machine learning algorithms (GLM, Elastic Net, GBM, SVM, KNN, RF, Naive Bayes, stepLDA, Logit, and PLS) was applied to refine the selection of genes closely related to prostate cancer (Passera et al., 2021). Differential expression analysis was conducted on these genes, with those exhibiting p < 0.05 and a fold change in expression ≥2 being identified as differentially expressed genes. Subsequently, single-gene survival regression analyses (OS/PFI/DSS/DFI) were performed on the core differentially expressed genes identified.

Furthermore, the expression landscape of NOP58 in prostate cancer was thoroughly investigated, demonstrating significant findings across various analyses. Immunohistochemical staining revealed marked NOP58 protein presence in prostate cancer tissues compared to adjacent non-cancerous tissues (Yu et al., 2004). The core gene interaction network highlighted NOP58’s central role (Papasaikas et al., 2015; Cervantes et al., 2020). Predictive models showed good calibration for prostate cancer prediction using NOP58 expression (Kearns and Lin, 2017). Differential expression analysis indicated significant upregulation of NOP58 in tumor tissues in both non-paired and paired samples (Sanchez-Palencia et al., 2011). The ROC curve demonstrated high diagnostic accuracy for NOP58 in distinguishing tumor from normal tissues. Further analysis revealed no significant expression differences across molecular subtypes but highlighted differences in immune subtype distributions and treatment outcomes correlated with NOP58 expression. Correlation analysis with CD274 and survival prognosis analyses indicated significant interactions and stratified survival outcomes. Univariate and multivariate Cox regression analyses showed NOP58’s significant impact on survival outcomes, with restricted cubic spline analysis exploring potential non-linear risk relationships. GSEA/GSVA enrichment analyses provided insights into metabolic pathways associated with NOP58 expression.

The discovery of NOP58 as a key regulatory factor provides a new perspective on the biology of prostate cancer and highlights its potential as a prognostic biomarker and therapeutic target (Arriaga-Canon et al., 2018; Felgueiras et al., 2014). The main purpose of this study is to deepen the understanding of the molecular mechanisms of prostate cancer by focusing on the SUMOylation pathway and its prognostic significance (Sun et al., 2023; Vlachostergios and Papandreou, 2012).

## Materials & methods

### Identification and analysis of prognostic genes in prostate cancer related to SUMO ubiquitination modifications

In the current study, we used bioinformatics methods to screen prognostic genes associated with SUMO ubiquitin modifications in prostate cancer (Sun et al., 2023; Zhang et al., 2023a). At the outset, prostate cancer transcriptome data extracted from The Cancer Genome Atlas (TCGA) database Successive systematic scale OS/PFI/DSS survival prognosis analyses were carried out to discover mRNAs closely related to prostate cancer survival outcomes (Reyes et al., 2021; Mu et al., 2020). The VennDiagram. I Identify overlap genes of OS-PFI-DSS; R package Next, these overlapping prognostic genes were further identified by binary Boruta algorithm to identify important PCa relevant genes (Liu et al., 2020; He et al., 2019). Furthermore, a panel of ten machine learning algorithms (GLM, Elastic Net, GBM, SVM, KNN, RF),m Naïve Bayes, stepLDA, Logit and PLS) was used to further narrow down genes closely associated with prostate cancer (Saeedi et al., 2022; Ying et al., 2021). Differential expression analysis was conducted on these genes, with those exhibiting p < 0.05 and a fold change in expression ≥2 being identified as differentially expressed genes. Subsequently, single-gene survival regression analyses (OS/PFI/DSS/DFI) were performed on the core differentially expressed genes identified. The forestplot package was used to create forest plots displaying hazard ratios and their 95% confidence intervals. Furthermore, a survival prognosis model was developed based on the expression profiles of these core differentially expressed genes, and survival prognosis curves were generated (Li et al., 2021; Wang et al., 2021). This process aimed to develop a diagnostic model for prognostic genes in prostate cancer related to SUMO ubiquitination modifications (Sun et al., 2023; Zhang et al., 2023a). Immunohistochemical data was sourced from the HPA database (https://www.proteinatlas.org/).

### GSEA and immune infiltration analysis

To perform differential analysis between tumor and normal groups, the limma package was utilized to compute the log2 fold change (log_2_FC) for each gene. Genes were ranked based on their log_2_FC values, and gene set enrichment analysis (GSEA) was carried out using the clusterProfiler package, focusing on the SUMO gene set. The enrichment score (ES) for each gene set was calculated, followed by significance and multiple hypothesis testing on these ES values. Additionally, the pROC package was employed for receiver operating characteristic (ROC) analysis to determine the 95% confidence interval, total area under the curve, and to plot a smooth ROC curve. This was done to assess the diagnostic performance of ssGSEAscore expression in both tumor and normal groups. The survival package facilitated Kaplan-Meier survival analysis, determining optimal cutoff values for high and low ssGSEAscore groups using the survminer package (ensuring a minimum proportion of 0.3 for both groups). The significance of the differences between high and low scoring groups was evaluated using the log-rank test with the survfit function. Univariate Cox survival analysis results were meta-analyzed via the inverse variance method, using log hazard ratio (HR) values as the primary measure. Statistical analyses and visualizations were conducted using R (version 4.3.2) with the Meta package.

### Prostate cancer expression landscape analysis

The expression levels of the core gene NOP58 in prostate cancer tissues and adjacent non-cancerous tissues were investigated using the Human Protein Atlas (HPA). To filter protein-protein interaction data, the ComPPI database was utilized, ensuring the exclusion of biologically implausible interactions and introducing interaction scores to quantify data accuracy. The diagnostic performance of gene expression in distinguishing tumor from normal tissue was assessed using ROC analysis via the pROC package, calculating the 95% confidence interval and AUC, and plotting ROC curves. The data used originated from TCGA-corrected RNA-seq data, processed through Firehose and normalized. Z-score standardization identified outliers, and the Wilcoxon Rank Sum Test assessed expression differences between tumor and normal tissues. Combining GTEx and TCGA data, Z-score standardization was again performed to exclude outliers, followed by ROC analysis to evaluate gene expression’s diagnostic performance. The Wilcoxon Rank Sum Test was also applied to compare NOP58 expression between prostate cancer and adjacent tissues. To evaluate the accuracy of model predictions, calibration curves and goodness-of-fit tests were employed. Six molecular immune subtypes related to tumor characteristics and prognosis were categorized by median value, and their significance in subtype proportions was assessed using the chi-square test. The Kruskal-Wallis Rank Sum Test compared NOP58 expression differences across various molecular subtypes. Clinical variables were statistically grouped based on median expression, and their proportions were evaluated using the chi-square test.

### Prostate cancer WGCNA analysis

Genes that exhibit similar expression patterns may be co-regulated, functionally related, or part of the same pathway. To identify hub genes and investigate the relationship between gene networks and specific phenotypes, we utilized Weighted Gene Co-expression Network Analysis (WGCNA). By employing the “WGCNA” package in R, we constructed a weighted gene co-expression network characterized by approximate scale-free properties. The analysis determined highly co-expressed genes through the correlation of their expression values. Topological overlap measurement (TOM) was used to generate network modules, and co-expression gene modules were identified via the dynamic hybrid cutting method, which is a bottom-up approach. Modules with dissimilarity thresholds lower than 0.25 were subsequently merged. The correlation between genes and modules was assessed by calculating gene significance (GS) and module significance (MS).

### Survival prognosis analysis

In prostate cancer tissue samples, the Pearson correlation between the target gene and both mRNA and miRNA was computed and represented using scatter plots. Only results where the absolute value of the correlation coefficient exceeded 0.3 were considered significant. Gene expression levels were categorized based on their correlation strength with the target gene into four groups: positive correlation, moderate correlation, weak correlation, and negative correlation, which were then visualized using a contingency table heatmap. Statistical significance was assessed using Fisher’s exact test. To examine the relationship between gene expression levels and patient survival, Kaplan-Meier survival analysis was employed. Detailed survival data analysis was conducted with the survival package in R, and the survminer package was used to determine optimal cutoff values for high and low expression groups, ensuring that each group contained at least 30% of the total sample size.

The survfit function was utilized to conduct log-rank tests on various survival metrics, including overall survival (OS), disease-specific survival (DSS), progression-free survival (PFS), progression-free interval (PFI), disease-free survival (DFS), and disease-free interval (DFI), to evaluate the significance of differences in survival between different gene expression level groups. Additionally, a meta-analysis using the univariate Cox proportional hazards model was performed, integrating results from multiple studies through the inverse variance method, with hazard ratio (HR) as the main measure of effect size to distinguish potential tumor-suppressive and tumor-promoting effects. Although this method categorizes genes effectively, it does not explore their biological mechanisms. Statistical analyses and visualizations were executed in the R (version 4.3.2) environment using the Meta package, which offers comprehensive functions for conducting meta-analyses and creating forest plots and funnel plots to visually present combined effect sizes and assess publication bias.

### Enrichment analysis

In this study, we employed a stratified approach to categorize samples into high and low gene expression groups, with the top 30% of samples designated as the high-expression group and the bottom 30% as the low-expression group. This method allowed us to identify the most significant changes in gene expression associated with disease progression. Additionally, GSEA was conducted using the fgsea function in the fgsea package, based on the KEGG database. Enrichment scores were calculated for gene sets, and those with an unadjusted p-value <0.05 and an adjusted p-value <0.25 were considered biologically significant. The results were visualized to highlight key biological processes. We redefined the 14 innate functions of tumor cells by projecting data onto multi-datasets and integrated datasets from CancerSEA, facilitating the identification of tumor cell states within a comprehensive functional framework. Functional state gene sets were calculated using the z-score algorithm proposed by Lee et al., implemented via the GSVA package in R, which transformed gene set values into z-scores. Pearson correlation analysis was then used to investigate the relationship between gene expression and functional states, specifically focusing on the correlation between gene expression and z-scores of gene sets. Finally, the gsva function in the GSVA package was employed to score 73 metabolic gene sets from the KEGG database. These GSVA scores were then compared between the high and low expression groups using the limma package to elucidate the roles of these pathways in disease progression.

### Immunotherapy sensitivity

To explore the relationship between gene expression and drug sensitivity, we conducted a non-parametric Spearman correlation analysis between gene expression levels and the area under the dose-response curve (AUC) values from the CTRP and PRISM databases. We also analyzed the relationship between gene expression and half-maximal inhibitory concentration (IC50) values from the GDSC1 and GDSC2 databases. A negative correlation indicated that high gene expression was associated with increased sensitivity to a drug, while a positive correlation suggested a gene’s high expression was linked to increased resistance to the drug. For potential novel therapeutic strategies, we assessed overlaps in cancer dependencies that could be mitigated by drug inhibition using cMAP analysis. The cMAP_gene_signatures RData file was utilized to establish the analysis framework. The XSum method was employed to compare gene features in signatures of the 150 most upregulated and downregulated genes with those in the cMAP database, calculating compound similarity scores. When gene expression had a repressive effect, the compounds were termed as TIPs. ROC analysis, performed using the pROC package, was used to evaluate how effectively these compounds could differentiate between immunotherapy responders and non-responders. The analysis included 95% confidence intervals, AUC values, and ROC curves to measure gene expression efficacy. Finally, Spearman correlation analysis was used to assess the relationship between gene expression and the TIP score, and autocorrelation of TIP scores was visualized using the linkET package. CYT levels were determined in the TCGA-HNSC dataset using the simpler package, and the Wilcoxon Rank Sum and Signed Rank tests were applied to examine differences in CYT scores between high and low PDCD1 expression groups.

### Core single gene immune infiltration analysis

Immune infiltration analysis was performed using data from the TIMER 2.0 database, which analyzes immune cell infiltration across TCGA samples. For the first time, we examined the infiltration of 10 representative types of immune cells in human pan-cancer tissues. The database employs various algorithms to estimate the quantities of individual immune cell types within the tumor microenvironment and their correlation with different gene expression levels. These algorithms, along with subsequent validation, enhance data quality and consistency, enabling a detailed investigation of the relationship between gene expression and immune cell infiltration. Correlation coefficients between gene expression and immune cell content were visualized using bar scatter plots to facilitate data interpretation, illustrating the relationship between gene expression and immune cell types. Based on the median level of gene expression, samples were divided into high-expression and low-expression groups. The Wilcoxon Rank Sum Test, a non-parametric method suitable for multiple data distributions, was applied to detect significant differences in immune cell content between the two groups. Significant immune cell types were further visualized with a heatmap, which arranged samples in ascending order of gene expression levels. The intensity of the heatmap colors provided an intuitive representation of immune cell content levels, revealing patterns and differences in immune infiltration among the samples.

### Core single gene genomics analysis

In this study, whole-genome CRISPR screening data from the DepMap portal were examined using the CERES algorithm to evaluate dependency scores for around 17,000 candidate genes. The pan-cancer mutation landscape of the core gene was visualized using the plotmafSummary function from the maftools package. To assess the independence between gene expression levels and specific gene mutation types, the independence_test function from the coin package in R was utilized, based on permutation tests. Genes with a mutation rate exceeding 10% and a p-value less than 0.01 were identified and visualized to highlight significant associations between gene expression and mutation types. For the tumor copy number spectrum analysis in the TCGA-HNSC project, genome copy number variations (CNVs) were identified using the gistic score method. The CNV profiles of 451 samples were visualized using bar plots, offering a clear representation of copy number changes across chromosomes. The quantitative metrics of genome alterations, such as FGA, FGG, and FGL, were defined and calculated based on the genomic distances of clonal regions. Analysis of variance (ANOVA) was conducted to investigate differences among specific gene expression subgroups, and if ANOVA was significant, multiple comparisons were performed using the TukeyHSD method to pinpoint specific group differences. The correlation between CNV scores and gene expression levels was analyzed using scatter plots combined with the Spearman rank correlation coefficient to measure the monotonic relationship between the two variables. Experimental data for copy number spectra were sourced from the TCGA Genome Characterization Center and obtained through whole-genome microarray measurements. Gene-level copy number estimates were derived using the TCGA FIREHOSE pipeline and the GISTIC2 method. The Kruskal-Wallis test, a non-parametric method for multiple sample comparisons, was used to compare gene expression differences among various copy number types (−2 to 2).

### Single gene pan-cancer single-cell sequencing analysis

In this study, single-cell gene expression data for prostate cancer were sourced from the GEO database, specifically dataset GSE172301. Heatmaps created with the pheatmap package effectively illustrated single-cell gene expression patterns across various cancer types. Hierarchical clustering analysis, using Euclidean distance and Ward’s minimum variance method, was employed to uncover intrinsic patterns of gene expression and their conservation among different cancers. Additionally, UMAP technology was utilized to explore expression patterns in high-dimensional data, maintaining the original data topology while reducing dimensions. UMAP analysis of CENPF gene expression data provided an intuitive display of gene expression patterns and facilitated the identification of key biological differences. To evaluate specific gene expression differences among various cell types, the Kruskal-Wallis Rank Sum Test was employed. This non-parametric statistical method is suitable for non-normally distributed samples and is effective in detecting significant differences among multiple independent sample groups. Moreover, AUCell scoring, which indicates pathway activity heterogeneity in cells, was dimensionally reduced and visualized using UMAP technology. This application of UMAP enabled an intuitive understanding of the distribution of these pathway activities and the identification of potential biological differences.

### Core gene single-cell spatial transcriptomics analysis

In this study, single-cell gene expression data for prostate cancer were sourced from the TISCH database. The pheatmap package was utilized to generate heatmaps, effectively revealing gene expression patterns at the single-cell level across various cancer types. Hierarchical clustering analysis, employing Euclidean distance and Ward’s minimum variance method, uncovered intrinsic gene expression patterns and their conservation across different cancers. Additionally, UMAP technology was applied to explore high-dimensional expression patterns, preserving the original data topology while reducing dimensions. This UMAP analysis of CENPF gene expression data provided an intuitive display of gene expression patterns and facilitated the identification of key biological differences.

To evaluate specific gene expression differences among various cell types, the Kruskal-Wallis Rank Sum Test was employed. This non-parametric statistical method is suitable for non-normally distributed samples and effectively detects significant differences among multiple independent sample groups. Furthermore, AUCell scoring, which indicates pathway activity heterogeneity in cells, was dimensionally reduced and visualized using UMAP technology. This approach allowed for an intuitive understanding of the distribution of pathway activities and the identification of potential biological differences.

### Cell culture

We obtained human cell lines PC-3 and LNCaP from the Shanghai Cell Bank (Shanghai, China). Each cell line was cultured under specific conditions to ensure optimal growth and viability. The PC-3 and LNCaP cell lines were maintained in RPMI 1640 medium, supplemented with 10% fetal bovine serum (FBS) to provide essential nutrients and hormones that promote cell proliferation. Additionally, 1% L-glutamine was added to the medium to support protein synthesis and maintain cellular metabolism. To prevent bacterial contamination, 1% penicillin–streptomycin solution was included. The cells were incubated at 37°C in a 5% CO_2_ atmosphere. All cell lines were regularly monitored for confluency and morphology under a microscope, and the media was changed every 2–3 days to maintain a fresh supply of nutrients and remove waste products. Cell passages were performed at 70%–80% confluency to avoid overgrowth and to maintain the cells’ physiological state. Trypsin-EDTA solution was used for cell detachment during passaging, and cells were counted using a hemocytometer to ensure accurate seeding densities for subsequent experiments.

### Cell proliferation assay

To evaluate cell proliferation rates, we employed the CCK-8 assay using the CCK-8 kit (Dojindo, Kumamoto, Japan). Cells were seeded into 96-well plates and cultured for 0, 24, 48, 72, and 96 h. Every day, a CCK-8 solution was added to each well and allowed to incubate with the cells for 2 hours. Cell viability was then assessed by measuring the absorbance at 450 nm using a microplate reader.

### Clone formation assay for cell proliferation

Cells in the logarithmic growth phase from each group were collected and diluted to a concentration of 500 cells/mL. To prepare the wells of a 6-well plate, 1 mL of medium was added to wet the wells, followed by the addition of 1 mL of the cell suspension to each well. Each group was plated in triplicate. The cells were incubated overnight at 37°C in a 5% CO_2_ incubator to allow for adhesion. After overnight incubation, cells were collected from each group, and 5 × 10^4^ cells per well were added to the corresponding wells, with the medium being changed every 2 days. Following a 12-day incubation period, the medium was discarded from the 6-well plate, and the wells were washed twice with PBS. To fix the cells, 1 mL of methanol was added to each well and left at room temperature for 20 min. After removing the methanol, 1 mL of 0.1% crystal violet was added to each well for staining, also at room temperature for 20 min. The wells were then washed with PBS until the background was clear. Colonies were photographed and counted.

### qRT-PCR

Total RNA was extracted from cells by adding 1 mL of Trizol reagent to each well and transferring the contents to 1.5 mL EP tubes, followed by a 10-min lysis. Next, 200 μL of chloroform was added to each tube, and the samples were centrifuged at 12,000 rpm for 15 min at 4°C. The upper aqueous phase was carefully transferred, and 400 μL of isopropanol was added. Following multiple rounds of centrifugation, the supernatant was discarded, and the RNA pellet was dissolved in 20 μL of DEPC-treated water. Reverse transcription into cDNA was performed under the following conditions: 25°C for 5 min, 50°C for 15 min, 85°C for 5 min, and 4°C for 10 min. The resulting cDNA was diluted 10-fold and then amplified using real-time fluorescent quantitative PCR, with GAPDH serving as the reference gene.

### Statistical analysis

The findings are based on a minimum of three independent experiments and are expressed as the mean ± standard deviation. Differences between groups were evaluated using either one-way analysis of variance (ANOVA) or Student’s t-test. A p-value of less than 0.05 was considered statistically significant, while a p-value of less than 0.01 indicated high statistical significance.

## Results

### Identification of SUMO ubiquitination-related prognostic genes in prostate cancer

**FIGURE 1:**
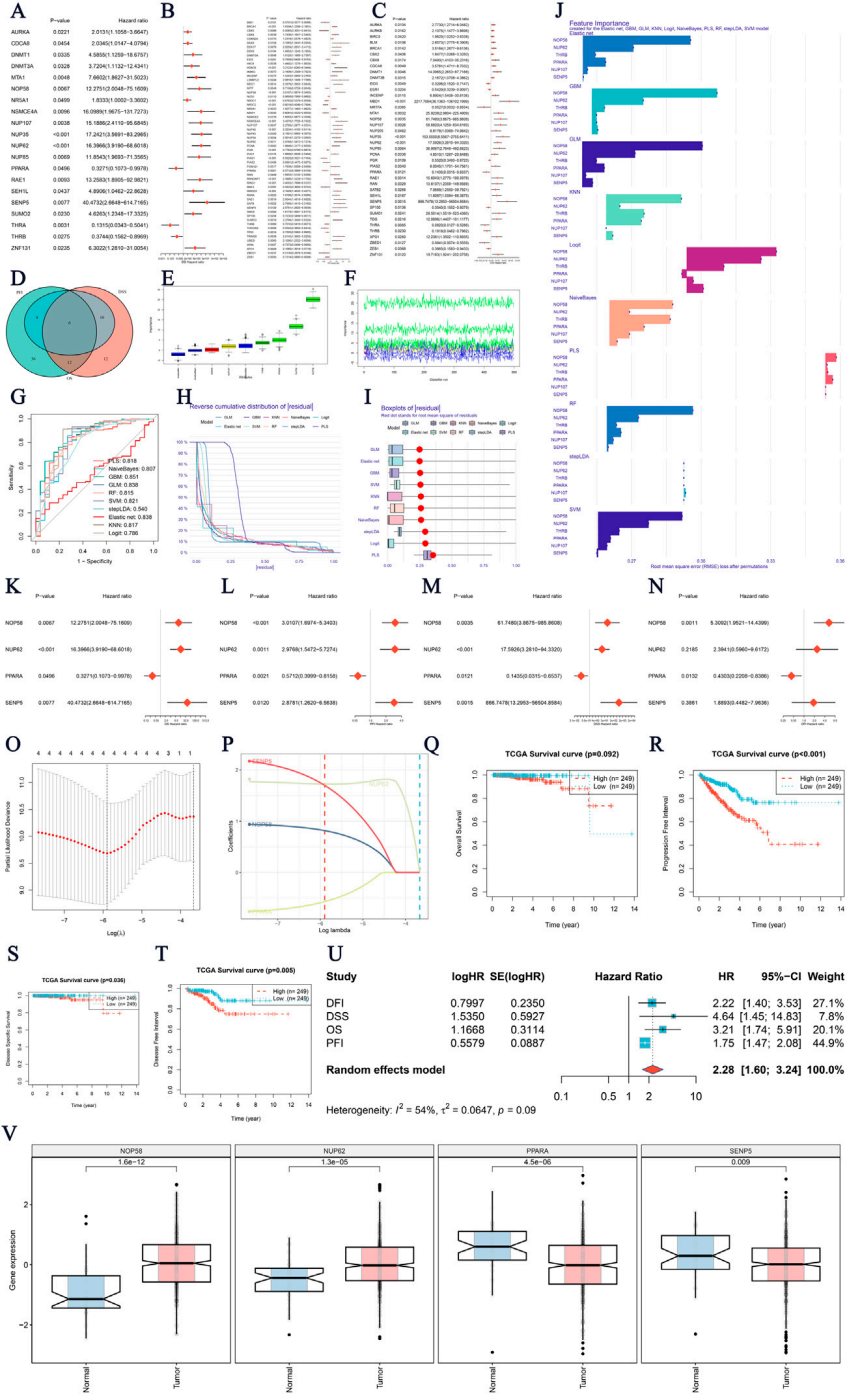
Identification of SUMO Ubiquitination-Related Prognostic Genes in Prostate Cancer **(A–C)** Survival prognostic genes related to Overall Survival (OS), Progression-Free Interval (PFI), and Disease-Specific Survival (DSS). The plots display hazard ratios (HRs) and confidence intervals (CIs) for each gene analyzed in relation to its predictive value. **(D)** Venn diagram showing the overlap of significant genes for OS, PFI, and DSS, where common prognostic genes represent overlapping survival metrics. **(E, F)** Boruta analysis identifying key genes. The boxplot compares feature importance scores, illustrating the significance of each gene identified by Boruta in constructing the prognostic model. **(G–J)** Screening of significant genes using 10 machine learning models. These bar charts highlight the top genes selected by each model. **(K–N)** Univariate Cox regression analysis for gene-based prognosis. Forest plots and prognostic genes for overall survival are presented. **(O–P)** Evaluation of the multigene survival model through calibration plots and receiver operating characteristic (ROC) curves, assessing the model’s ability to discriminate patient outcomes. **(Q–T)** Kaplan-Meier survival curves for OS, PFI, DSS, and Disease-Free Interval (DFI), illustrating survival probabilities for patients grouped by the multigene model, with log-rank p-values indicating significance levels. **(V)** Expression analysis of four core genes in prostate cancer. Boxplots show the differential expression in tumor versus normal tissues, suggesting their potential as novel diagnostic/therapeutic targets. P-values indicate statistical significance.

In this study, we identified key prognostic genes associated with SUMO ubiquitination modifications in prostate cancer. First, we examined genes linked to OS, PFI, and DSS (Figures 1A–C). To refine the analysis, a Venn diagram (Figure 1D) was used to identify the intersection of genes associated with OS, PFI, and DSS, allowing us to pinpoint common genes that are prognostic across multiple survival metrics. Further refinement using the Boruta algorithm (Figures 1E, F) identified key genes, with boxplots and feature importance scores highlighting those deemed critical for the prognostic model. Next, we employed ten machine learning models to screen for genes significantly associated with prostate cancer prognosis (Figures 1G–J). The bar charts illustrate the top genes selected by different models, showcasing their frequency of selection and importance in predicting prognosis. Univariate Cox regression analysis was subsequently conducted (Figures 1K–N), with forest plots displaying the HRs and CIs of these genes, reflecting their independent impacts on prognosis. Calibration plots and receiver operating characteristic (ROC) curves for the model’s predictions of patient outcomes are shown in Figures 1O, P. Kaplan-Meier survival curves for OS, PFI, DSS, and DFI were generated based on the multigene model groups, with log-rank p-values indicating statistically significant differences between patient groups. Lastly, a detailed Cox regression analysis for the multigene model was performed (Figure 1U). The forest plot effectively summarizes the HRs and corresponding CIs for the multigene model, demonstrating its comprehensive prognostic significance across various survival measures. Validation methods were combined with boxplots (Figure 1V) to compare, in a straightforward manner, the expression levels of the core genes in normal versus tumor tissues.

### NOP58 GSEA immune infiltration analysis and prognostic evaluation

**FIGURE 2:**
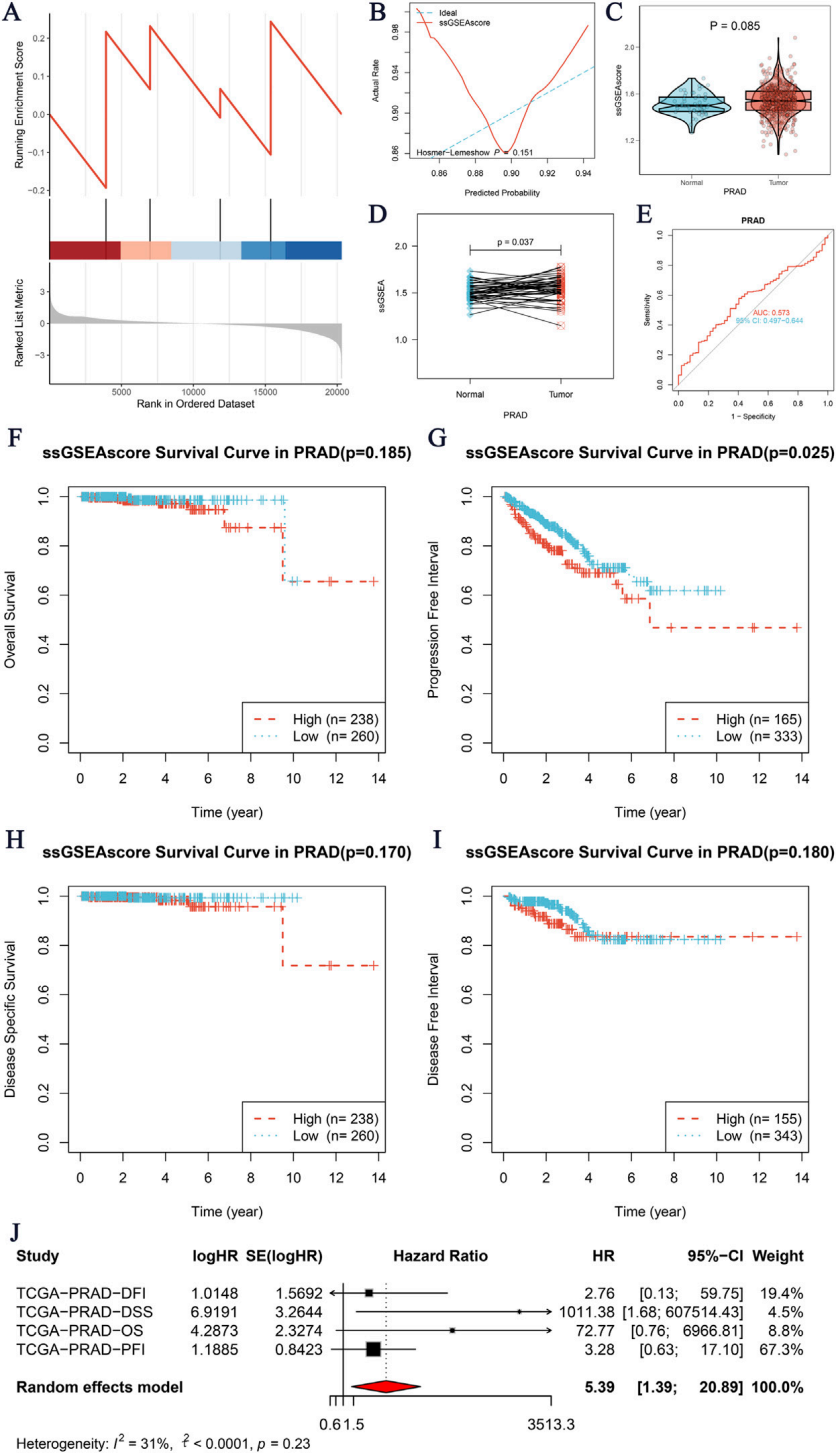
Core Gene GSEA Immune Infiltration Analysis. **(A)** GSEA enrichment analysis of the core gene SMUO gene set. The *x*-axis represents the rank in the ordered dataset, and the *y*-axis shows the enrichment score. **(B)** Calibration curve and goodness-of-fit test for the prediction of tumor versus normal groups using ssGSEAscore expression. The *x*-axis indicates the predicted probability, and the *y*-axis shows the actual rate, with the ideal curve as a reference. **(C, D)** Expression differences of ssGSEAscore between tumor and normal groups. **(C)** Non-matched samples are shown using a violin plot, with statistical significance (P = 0.085). **(D)** Matched samples are shown with a paired analysis plot, indicating a significant difference (P = 0.037). **(E)** ROC curve evaluating the diagnostic performance of ssGSEAscore for distinguishing between tumor and normal groups. The area under the curve (AUC) is provided, demonstrating the model’s discriminatory ability. **(F–I)** Kaplan-Meier survival analyses for four survival periods: Overall Survival (OS) **(F)**, Progression-Free Interval (PFI) **(G)**, Disease-Specific Survival (DSS) **(H)**, and Disease-Free Interval (DFI) **(I)**. The survival curves compare high (red) and low (blue) ssGSEAscore groups, with the number of patients (n) indicated for each group and the corresponding p-values. **(J)** Meta-analysis of survival hazard ratios, presenting logHR and 95% confidence intervals for different survival outcomes, including DFI, DSS, OS, and PFI.

Our study aimed to investigate the core gene NOP58 and its role in immune infiltration within the tumor microenvironment. The Gene Set Enrichment Analysis (GSEA) revealed significant enrichment of the core gene NOP58 in the SUMO gene set (Figure 2A). The predictive performance of the ssGSEAscore was evaluated using a calibration curve and goodness-of-fit test, showing an acceptable fit between the predicted and observed probabilities for distinguishing between tumor and normal groups (Figure 2B). Further analysis comparing the expression levels of ssGSEAscore between tumor and normal samples revealed significantly higher expression in tumor samples, both in non-matched (P = 0.085, Figure 2C) and paired sample analyses (P = 0.037, Figure 2D). The diagnostic efficacy of ssGSEAscore, assessed using ROC curve analysis, exhibited a high area under the curve (AUC) value, indicating excellent discriminatory power in differentiating tumor from normal samples (Figure 2E). Kaplan-Meier survival analysis demonstrated the prognostic significance of ssGSEAscore in OS, PFI, DSS, and DFI, with lower survival rates observed in the high ssGSEAscore group and the most significant difference noted in PFI (P = 0.025, Figures 2F–I). A meta-analysis of survival hazard ratios further consolidated these findings, indicating a significantly higher hazard ratio for adverse outcomes in the high ssGSEAscore group compared to the low group (Figure 2J). The analysis included hazard ratios for DFI, DSS, OS, and PFI, with heterogeneity statistics showing moderate variability among the studies. Collectively, these results highlight the critical role of NOP58 and ssGSEAscore in tumor progression and prognosis, underscoring their potential as biomarkers for cancer diagnosis and therapeutic targets.

### The critical role of NOP58 in prostate cancer and its association with SUMOylation modifications

**FIGURE 3:**
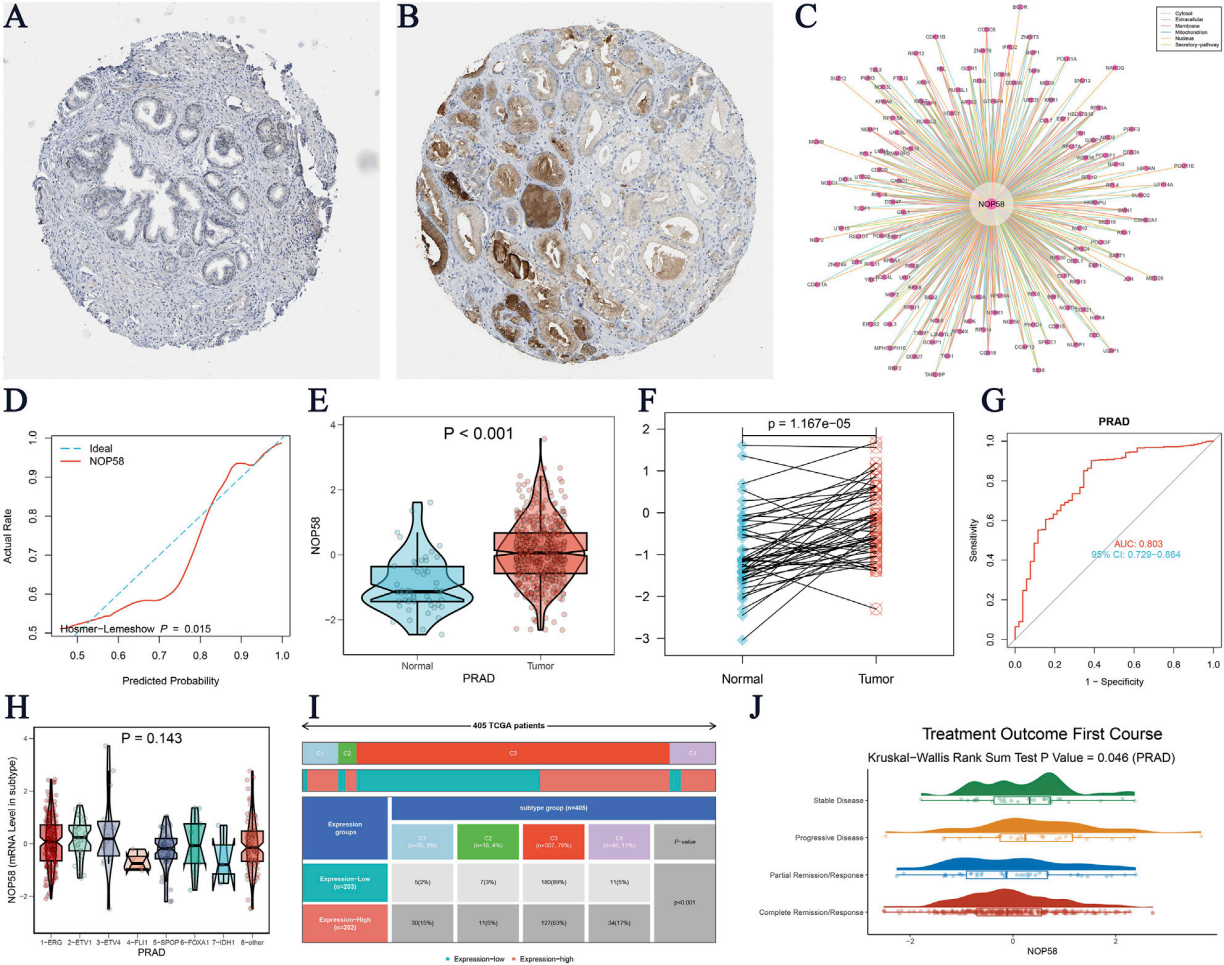
Comprehensive Analysis of NOP58 Expression in Prostate Cancer **(A–B)** Immunohistochemical staining for NOP58 in prostate cancer and adjacent non-cancerous tissues. **(A)** Adjacent non-cancerous tissue shows lower NOP58 staining, while **(B)** prostate cancer tissue displays significantly higher NOP58 staining (data from the HPA database). **(C)** Interaction network of NOP58, highlighting experimentally validated and predicted protein interactions, emphasizing NOP58’s central role. **(D)** Calibration plot assessing the predictive accuracy of the NOP58 expression model in prostate cancer. **(E–F)** Differential expression analysis of NOP58 in prostate cancer. **(E)** Violin plot reveals significant upregulation of NOP58 in unpaired tumor samples compared to normal tissues (p < 0.001). **(F)** Paired sample analysis shows consistent upregulation in tumor tissues (p = 1.157e-05). **(G)** ROC curve evaluating NOP58’s diagnostic performance in distinguishing tumor from normal tissues, with the area under the curve (AUC) indicating high diagnostic accuracy. **(H)** Violin plot showing no significant difference in NOP58 expression across molecular subtypes of prostate cancer (p = 0.143). **(I)** Stacked bar chart showing immune subtype distribution in high and low NOP58 expression groups across cancers. **(J)** Violin plot illustrating the correlation between NOP58 expression and treatment outcomes after the first course of therapy (Kruskal-Wallis Rank Sum Test, p = 0.064).

**FIGURE 4:**
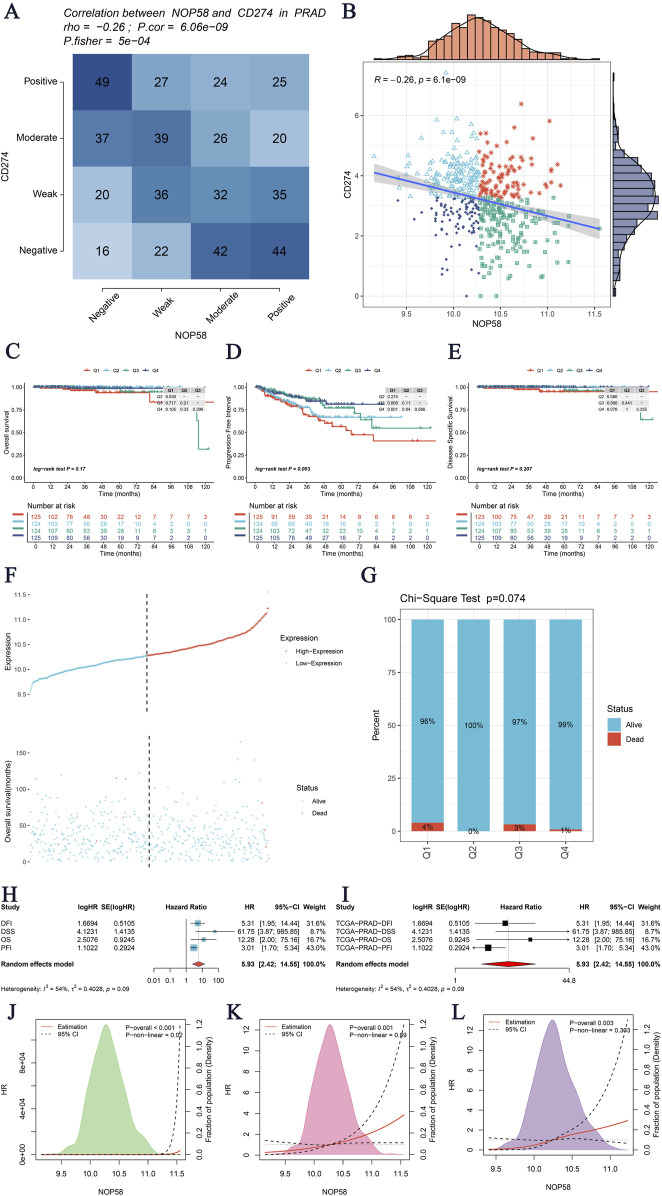
Analysis of NOP58 Gene Interactions and Survival Prognosis. **(A)** Correlation analysis of the NOP58 gene with CD274 in prostate adenocarcinoma (PRAD) using Fisher’s exact test. The heatmap displays the correlation between the expression levels of NOP58 and CD274, with statistical significance indicated by p-values. **(B)** Scatter plot illustrating the correlation between NOP58 and CD274 expression levels. The blue line represents the linear regression fit, with R and p-values indicating the strength and significance of the correlation, respectively. **(C–E)** Kaplan-Meier survival analysis for three survival metrics: Overall Survival (OS), Disease-Specific Survival (DSS), and Progression-Free Interval (PFI). The survival curves are stratified by NOP58 expression levels, with the number of patients at risk displayed below the curves. **(F–G)** Graphical representation of NOP58 gene expression in relation to patient survival status. Panel F shows the distribution of NOP58 expression levels with corresponding survival status (alive vs. dead). Panel G presents the Chi-Square test results for survival status across different quartiles of NOP58 expression, with the p-value indicated. **(H–I)** Univariate and multivariate Cox regression analyses of NOP58 gene expression. Hazard ratios (HR) with 95% confidence intervals (CI) are displayed, assessing the impact of NOP58 expression on survival outcomes. Panel H shows results from univariate analysis, while panel I shows multivariate analysis results adjusted for potential confounders. **(J–L)** Restricted cubic spline analysis to explore the potential non-linear relationship between NOP58 expression and the risk for OS, DSS, and PFI. The plots illustrate the risk estimates across a range of NOP58 expression levels, with confidence intervals indicated by dashed lines.

**FIGURE 5:**
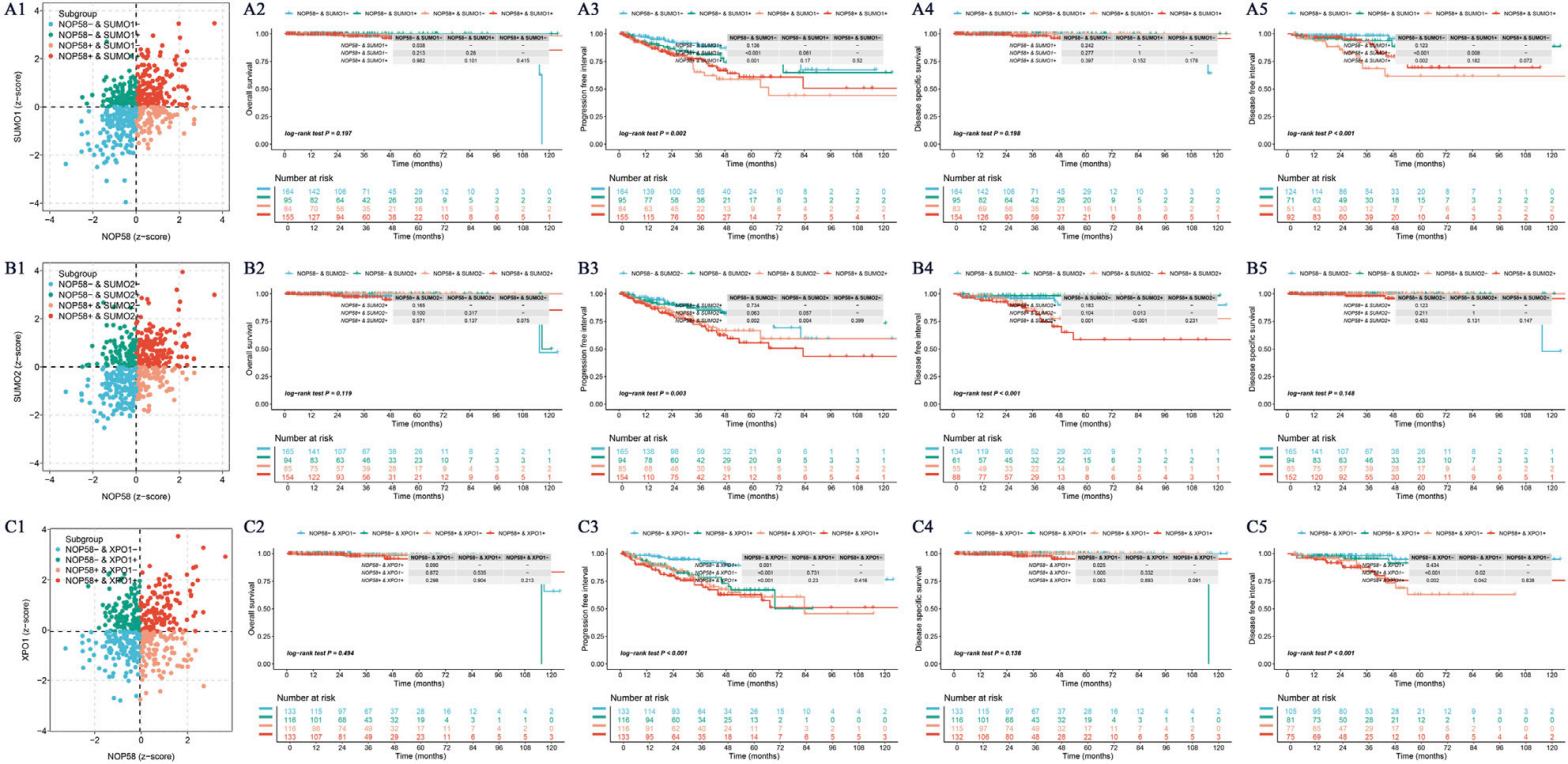
NOP58 Gene and SUMO-Related Gene Analysis. **(A)** NOP58-SUMO1 molecular subtype analysis and Kaplan-Meier survival curves. The scatter plot shows four molecular subtypes based on NOP58 and SUMO1 expression, with corresponding KM curves for survival probability. **(B)** NOP58-SUMO2 molecular subtype analysis. A similar scatter plot and KM analysis are presented for NOP58 and SUMO2. **(C)** NOP58-XPO1 molecular subtype analysis. KM survival curves demonstrate outcomes for patients stratified by NOP58 and XPO1 expression.

The expression landscape of NOP58 in prostate cancer was thoroughly investigated, revealing significant findings across various analyses. In the HPA dataset, immunohistochemical staining demonstrated a marked overexpression of NOP58 protein in prostate cancer tissues compared to adjacent non-cancerous tissues (Figures 3A, B). The core gene interaction network highlighted NOP58’s central role (Figure 3C). Predictive models showed good calibration for prostate cancer prediction using NOP58 expression (Figure 3D). Differential expression analysis indicated significant upregulation of NOP58 in tumor tissues in both non-paired (P < 0.001) and paired samples (P = 1.157e-05) (Figures 3E, F). The ROC curve demonstrated high diagnostic accuracy for NOP58 in distinguishing tumor from normal tissues (Figure 3G). Further analysis revealed no significant expression differences across molecular subtypes (P = 0.143), but it did highlight differences in immune subtype distributions and treatment outcomes correlated with NOP58 expression (Figures 3H–J). Correlation analysis with CD274 and survival prognosis analyses indicated significant interactions and stratified survival outcomes (Figures 4A–G). Univariate and multivariate Cox regression analyses demonstrated NOP58’s significant impact on survival outcomes, with restricted cubic spline analysis exploring potential non-linear risk relationships (Figures 4H–L). GSEA/GSVA enrichment analyses provided insights into metabolic pathways associated with NOP58 expression (Figures 5A–E). Additionally, double gene molecular subtype analysis with SUMO1, SUMO2, and XPO1 genes revealed survival outcome differences across molecular subtypes, further emphasizing NOP58’s critical role in prostate cancer biology and patient prognosis (Figures A1–C5). Additionally, double gene molecular subtype analysis with SUMO1, SUMO2, and XPO1 genes revealed survival outcome differences across molecular subtypes, further emphasizing NOP58’s critical role in prostate cancer biology and patient prognosis (Figures A1–C5). These findings suggest that SUMO modifications may play a pivotal role in prostate cancer progression and prognosis. This comprehensive analysis underscores the importance of NOP58 as a prognostic biomarker and potential therapeutic target in prostate cancer.

### Immune function and pathway enrichment analysis of NOP58 in prostate cancer

**FIGURE 6:**
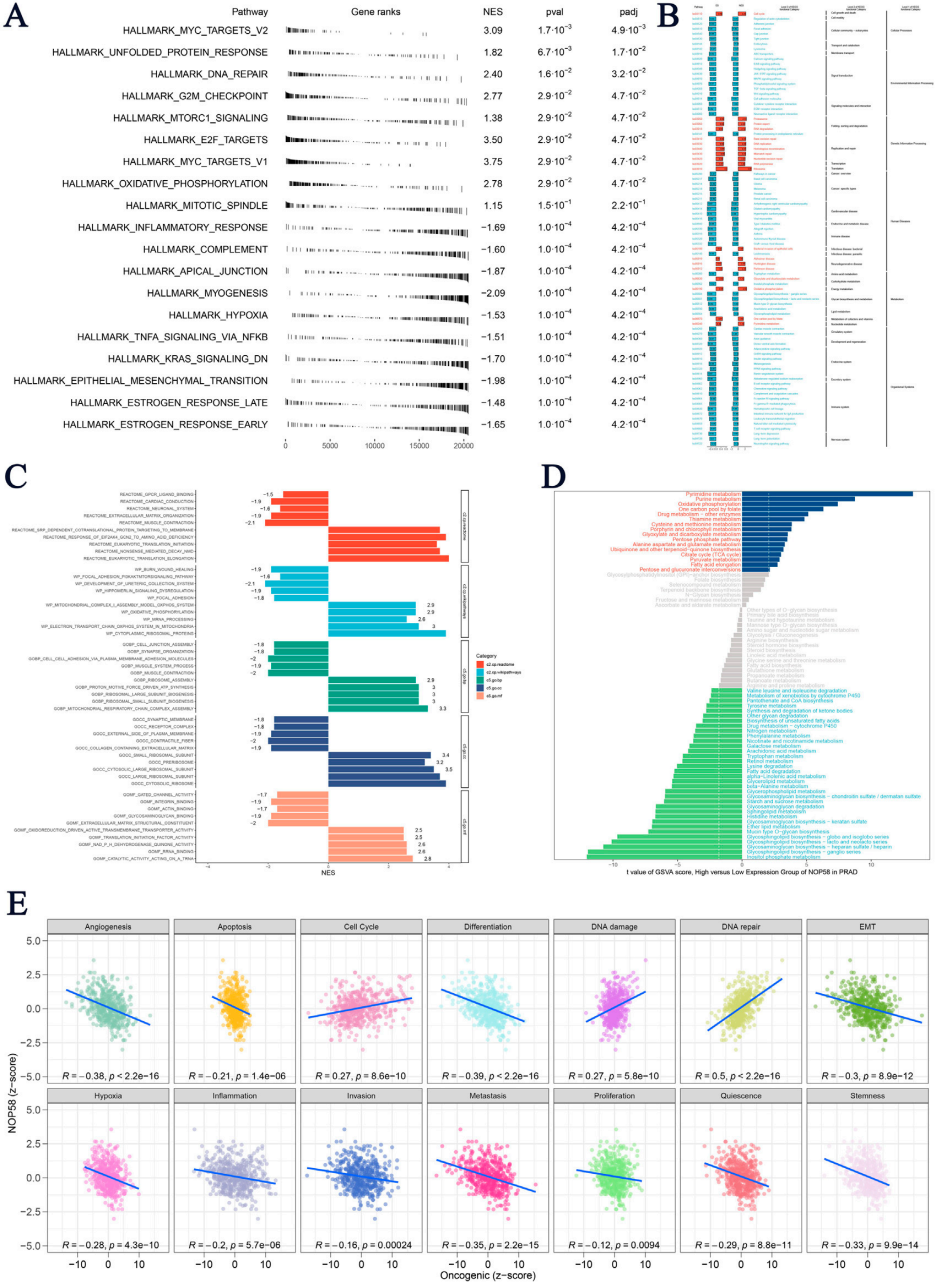
NOP58 Gene Immune Function Analysis **(A)** Hallmark Gene Set Enrichment Analysis (GSEA) for high and low NOP58 expression groups, based on hallmark gene sets, listing normalized enrichment scores (NES), p-values, and FDR q-values. **(B)** KEGG pathway enrichment analysis comparing enriched pathways between high and low NOP58 expression groups. **(C)** ClusterProfiler-based GSEA showing enrichment scores for gene sets in NOP58 high vs. low expression groups. **(D)** GSVA score comparison of metabolic pathways between NOP58 expression groups, with significant pathways highlighted. **(E)** Pearson correlation analysis of NOP58 expression and tumor states using GSVA scores, showing correlations across 14 tumor states.

In conclusion, these findings highlight NOP58 as a master regulator in metabolic adaptation, immune regulation, and tumor suppression pathways in prostate cancer, underscoring its potential as a biomarker. The hallmark GSEA presented in Figure 6A for the high expression group of NOP58 revealed several key pathways, including MYC_TARGETS_V2 (NES = 3.09, P = 1.7e-03, FDR = 4.9e-03) and UNFOLDED_PROTEIN_RESPONSE. Key pathways related to DNA repair (NES = 2.40) and the G2/M checkpoint (NES = 2.77) also showed strong enrichment in the high expression group of NOP58, suggesting significant associations between NOP58 transcription levels and processes such as cell division and DNA damage response. Figure 6B presents the KEGG pathway enrichment analysis comparing pathways that differ between high and low NOP58 expression groups. Figure 6C illustrates the enrichment scores for various gene sets using ClusterProfiler-based GSEA. High expression of NOP58 was significantly enriched in genes related to oxidative phosphorylation and immune response, suggesting a potential regulatory role of NOP58 in metabolism and immunity in PCa. The MSI GSVA score analysis (Figure 6D) also confirmed the above results, highlighting significant metabolic pathways. Signaling pathways and key metabolic processes, such as oxidative phosphorylation and purine metabolism, were enriched in the high expression group, further supporting NOP58 as a key factor driving metabolic adaptation and DNA repair. Pearson correlation analysis revealed that NOP58 expression was significantly negatively correlated with angiogenesis (R = −0.38), apoptosis (R = −0.21, p = 1.4e-06), and metastasis (R = −0.35, p = 2.2e-15) (Figure 6E). Additionally, NOP58 expression was significantly correlated with DNA repair (R = 0.5, p = 2.2e-16) and inversely correlated with quiescence (R = −0.29, p = 8.8e-11), traits typically associated with aggressive tumors.

### Immune microenvironment and immunotherapy sensitivity analysis of NOP58 in prostate cancer

**FIGURE 7:**
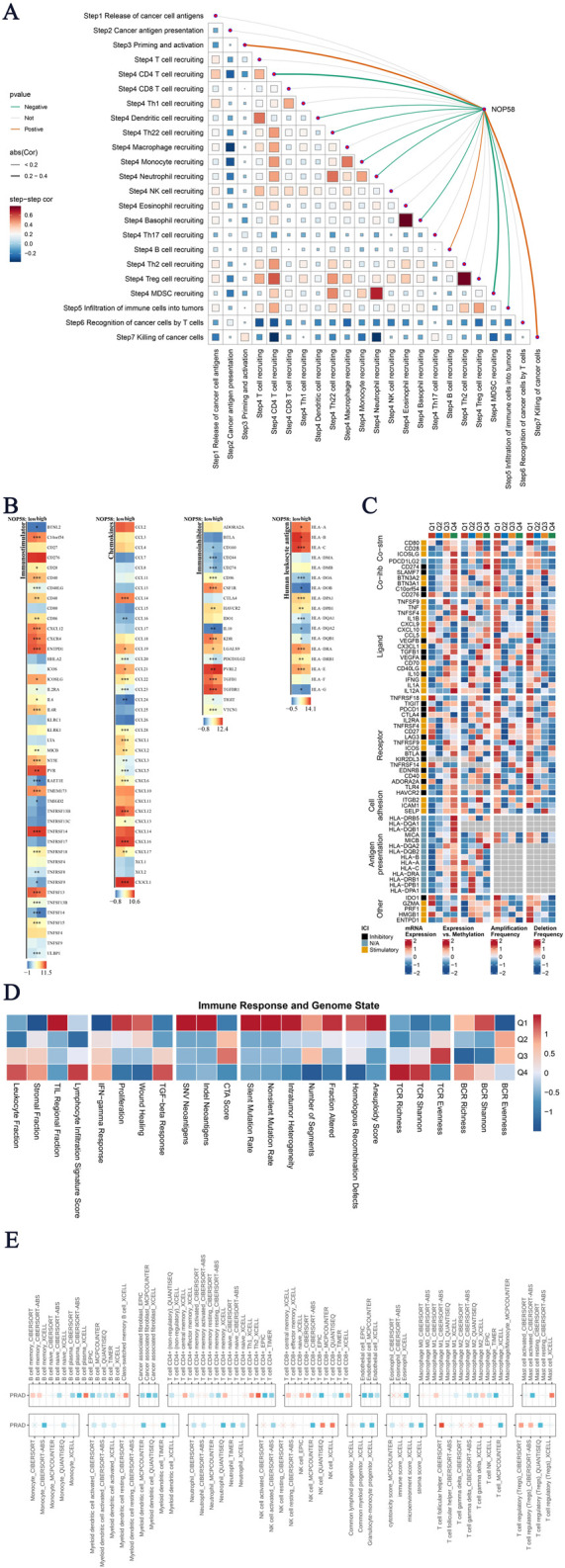
Analysis of Core Gene Immunotherapy Sensitivity **(A)** Tumor immune microenvironment (TME) scores correlated with NOP58 expression. The matrix shows Spearman correlations and auto-correlation of TME scores, with significant results highlighted. **(B)** Differentially expressed genes (DEGs) analysis showing immune stimulatory/inhibitory genes, chemokines, and HLAs between high and low NOP58 expression groups. Heatmaps display significant changes in expression levels. **(C)** Immunomodulator regulation analysis across stimulatory, inhibitory, and other genes based on NOP58 expression. **(D)** Heatmap illustrating the association between immune response markers and genomic status in different sample groups, showing relationships between genetic factors and immunity. **(E)** Genomic status and immune response correlation heatmap, showing the relationship between genomic events and immunogenic response outcomes.

In this study, we evaluated the association between NOP58 expression and the tumor immune microenvironment (TME) status, as well as its impact on immunotherapy sensitivity in prostate cancer. Subsequent analyses revealed a detailed series of results. A Spearman correlation analysis was performed to examine the relationship between NOP58 expression and various TME scores, as shown in Figure 7A. The results showed a strong association between NOP58 expression and immune pathways, particularly those involving antigen presentation, CD4+/CD8+ T cell recruitment, and immune cell infiltration. Specifically, NOP58 was involved in immune priming and T cell recognition, suggesting its potential role in modulating immune responses within the tumor microenvironment. In Figure 7B, we performed a differential expression analysis of immune-related genes, categorized as immune-stimulatory/inhibitory genes, chemokines, and HLA genes, comparing high and low NOP58 expression groups. Heatmaps demonstrated upregulation of chemokines and immune-stimulatory genes in the high NOP58 expression group, indicating a stronger immune response. Conversely, immune-inhibitory genes exhibited variable regulation by NOP58. We further extended our study to examine the regulation of immunomodulators based on NOP58 expression (Figure 7C). The results highlighted the influence of NOP58 on immune checkpoint regulation, which could in turn affect the response to immunotherapies. Figure 7D presents a heatmap analysis of immune response markers across different genomic statuses in the sample groups. Strong correlations were observed between different genomic alterations and immune-related factors, revealing the genetic origins of immune modulation in the tumor microenvironment. Figure 7E demonstrates the correlation between genomic events and immunogenic response outcomes. NOP58 expression levels and immune evasion mechanisms were preferentially enriched in mutated or CNA-related pathways, as highlighted in the heatmap. These correlations suggest previously unrecognized roles for NOP58 as a predictive marker for assessing the impact of immunotherapy in prostate cancer patients. Our analysis uncovered a critical role for NOP58 in shaping the tumor immune microenvironment and modulating immunotherapy responsiveness in prostate cancer. Through its role in modulating immune regulatory pathways and checkpoints, NOP58 emerges as a promising candidate for predicting patient response to immunotherapy. The results offer new insights into the potential for personalized anti-NOP58 immunotherapy in prostate cancer.

### Single-cell and spatial transcriptomics analysis of NOP58 in prostate cancer

**FIGURE 8:**
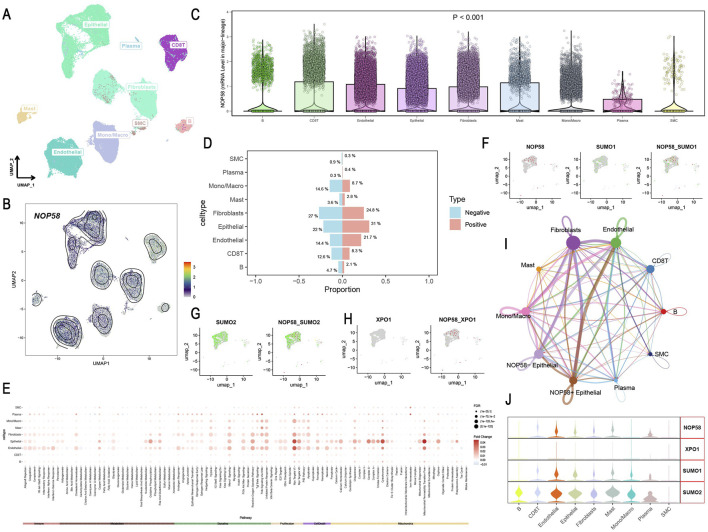
Single-Cell Sequencing Analysis of NOP58 in Prostate Cancer. **(A, B)** UMAP visualization of cell lineages at single-cell resolution. **(A)** UMAP plot showing cellular clusters; **(B)** contour lines show NOP58 expression levels. **(C)** Violin plots illustrating NOP58 expression across cell clusters, with statistical significance. **(D)** Bar graph showing proportions of NOP58-positive and NOP58-negative cells across different cell types. **(E)** Heatmap showing differential pathway enrichment between NOP58-positive and NOP58-negative cell types. **(F–H)** UMAP representations of NOP58 co-expression with SUMO1, SUMO2, and XPO1 genes, showing proportions across cell types. **(I)** Network plot illustrating communication between cell subgroups, with edges representing interactions and node sizes corresponding to interaction strength. **(J)** Violin plots showing the expression levels of NOP58, XPO1, SUMO1, and SUMO2 across different cell clusters.

Single-cell sequencing analysis revealed distinct cellular clusters within prostate cancer tissue, as visualized through UMAP, highlighting diverse cell lineages (Figure 8A). NOP58 gene expression was mapped, with contour lines indicating varying levels across different cells (Figure 8B). Differential expression analysis indicated significant overexpression of NOP58 in specific cell clusters, with violin plots showing distribution levels and statistical significance (p < 0.001) (Figure 8C). Proportional analysis of cell types between NOP58-positive and NOP58-negative groups showed distinct differences, with bar plots depicting percentages and error bars representing standard deviations (Figure 8D). Pathway analysis revealed differential pathway enrichment, displayed in a heatmap with color intensity indicating enrichment levels (Figure 8E). Co-expression studies demonstrated the correlation between NOP58 and SUMO1, SUMO2, and XPO1 genes, with scatter plots and heatmaps illustrating these relationships (Figures 8F–H). Network analysis of cell subgroups indicated extensive communication pathways, shown in a network plot with node sizes corresponding to interaction degrees (Figure 8I). Additionally, violin plots highlighted the heterogeneous expression of NOP58, XPO1, SUMO1, and SUMO2 across various cell types (Figure 8J).

### Spatial transcriptomics and drug sensitivity analysis of NOP58 in prostate cancer

**FIGURE 9:**
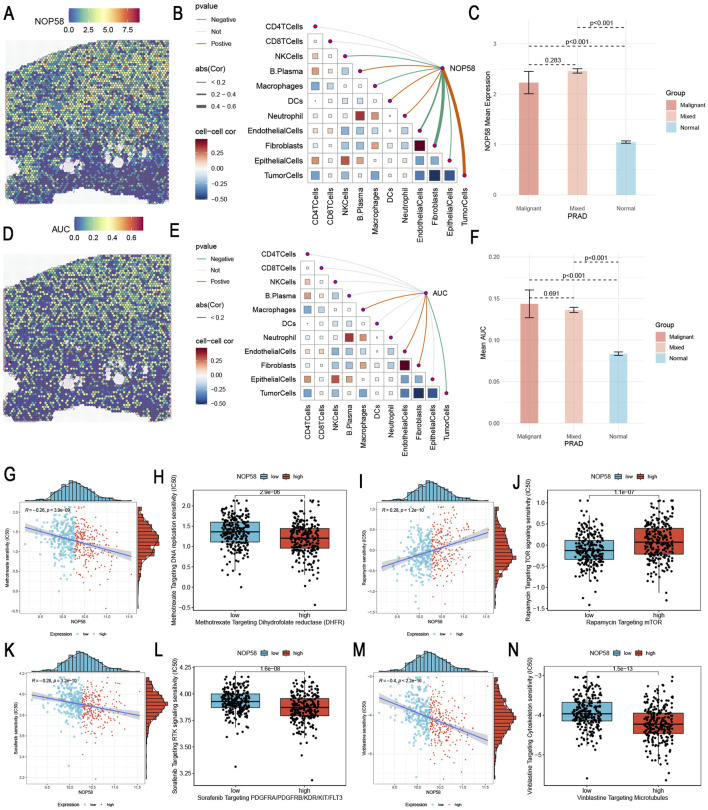
Spatial Transcriptomics Analysis of NOP58 in Prostate Cancer and NOP58 Gene Drug Sensitivity Analysis **(A)** Spatial localization of NOP58 single-gene expression within prostate cancer tissue. The heatmap shows the expression levels, with color gradients indicating the intensity of NOP58 expression across the tissue. **(B)** Spearman correlation analysis between NOP58 gene expression and various microenvironment components at single-cell resolution. The plot illustrates the relationship between NOP58 expression and cell types, including CD4^+^ T cells, CD8^+^ T cells, macrophages, fibroblasts, endothelial cells, and tumor cells. Correlation coefficients, represented by color intensity, reflect the strength of the associations. **(C)** Comparison of NOP58 expression levels across different microenvironments: malignant, mixed malignant, and normal. The bar graph shows the mean NOP58 expression for each group, with error bars denoting standard deviations. Statistical significance (p < 0.001) highlights distinct differences in expression between groups. **(D)** Spatial localization of NOP58 AUC scores within tissue sections. The heatmap displays AUC scores, with color gradients representing the intensity of NOP58 activity across the tissue, providing insights into its spatial distribution. **(E)** Spearman correlation between gene set AUC scores and microenvironment components at spatial resolution. The plot shows the correlations of various gene sets with different cell types in the microenvironment, with color intensity reflecting the strength of interaction. **(F)** Comparison of gene set AUC scores across malignant, mixed malignant, and normal microenvironments. The bar graph illustrates the mean AUC scores for each group, with error bars representing standard deviations. Significant differences (p < 0.001) highlight the variability in gene set activity across these environments. **(G–N)** Drug sensitivity analysis based on NOP58 expression levels. Each scatter plot represents individual samples, with the *x*-axis corresponding to NOP58 expression levels and the *y*-axis representing drug IC50 values. Samples are categorized into high-expression (red) and low-expression (blue) groups based on median NOP58 expression. High NOP58 expression is associated with increased sensitivity to specific drugs, indicating its potential influence on therapeutic response.

In this study, we identified the expression locus of NOP58 in prostate cancer tissues using spatial transcriptomics analysis, and subsequently examined its effect on drug resistance. The results indicate that NOP58 expression is associated with tumor microenvironment dynamics and therapeutic efficacy. Deconvolution of spatial transcriptomics data revealed the spatial distribution of distinct cell types in prostate cancer tissues. Notably, the localization of immune cells, macrophages, endothelial cells, and fibroblasts in the tumor microenvironment revealed distinct spatial arrangements among these cell types in different regions of the tumor (Supplementary Figure S1A). Figure 5 illustrates the spatial differences in gene expression within prostate cancer tissue, comparing malignant, normal, and mixed malignant/normal regions. Heterogeneity in gene expression within the tumor microenvironment was further demonstrated by relatively lower expression of certain or all of these targets in normal regions (Supplementary Figures S1B, C). Figure 9A shows the spatially segregated expression map of NOP58 in prostate cancer tissue sections. The heatmap illustrates differential expression levels, with elevated NOP58 expression enriched in specific areas of the tumor tissue. High expression levels are represented as hot spots in predominantly cold malignant areas using color gradients. We then performed Spearman correlation analysis to examine the association between NOP58 expression and various components of the TME at the single-cell level (Figure 9B). NOP58 expression was associated with immune cell populations, particularly CD4^+^ T cells, CD8^+^ T cells, macrophages, and fibroblasts. In contrast, weaker correlations were found between NOP58 and endothelial cells or tumor cells, likely due to NOP58-mediated immune cell regulation in the PCa microenvironment. As shown in Figure 9C, NOP58 was overexpressed in malignant tissues compared to normal and mixed malignant tissues (p < 0.001). The bar chart illustrates significant differences in mean expression levels between malignant and normal tissues, with the highest NOP58 expression observed in malignancies. This supports NOP58’s role in tumor development and its potential diagnostic utility for cancer status. Figure 9D depicts the spatial distribution of NOP58 activity across multiple tissue sections. Similarly, expression data revealed a consistent trend: high AUC scores were typically observed in regions with high NOP58 expression. This spatial distribution may provide insight into the biological and functional roles of NOP58 in specific tumor regions, suggesting potential differences in disease progression or therapeutic response. The strongest correlation with NOP58 expression was observed in gene sets related to immune responses, particularly T cell activation and fibroblast recruitment (Figure 9E). This suggests that NOP58 plays a role in critical immune functions within the prostate cancer microenvironment. AUC scores of NOP58-related gene sets were significantly elevated in the malignant microenvironment compared to mixed malignant and normal tissues (p < 0.001) (Figure 9F). The variation in NOP58 activity across different tissue types highlights its potential role as a mediator of tumor behavior. ROC curve analysis indicated that NOP58 expression could accurately differentiate responders from non-responders in various cancer types, including melanoma, NSCLC, and GBM, with AUC values exceeding 0.8 in some datasets. Thus, NOP58 may serve as a potential biomarker for predicting immunotherapy response (Supplementary Figure S1D). To analyze the correlation between NOP58 expression and drug sensitivity, Spearman correlation analysis was performed using the GDSC1 and GDSC2 databases (Supplementary Figures S1E, F). This suggests that NOP58 expression could influence drug response and may serve as a potential biomarker for predicting chemotherapy sensitivity (Supplementary Figures S1E, F). In our study, we investigated the relationship between NOP58 expression levels and drug sensitivity across several therapeutic agents targeting key pathways in cancer treatment. Our analysis revealed that high NOP58 expression was significantly correlated with increased sensitivity to several anticancer agents. For instance, high NOP58 expression was associated with lower IC50 values for Methotrexate targeting dihydrofolate reductase (DHFR), suggesting that NOP58 may influence the effectiveness of this drug (Figures 9G, H). Similarly, NOP58 overexpression led to enhanced sensitivity to Rapamycin, a known mTOR inhibitor, indicating potential therapeutic benefits in tumors with elevated NOP58 levels (Figures 9I, J). Other drugs, such as Sorafenib targeting PDGFR/RAF/VEGFR/RTKs (Figures 9K, L) and Venetoclax targeting microtubules (Figures 9M, N), also showed consistent trends, where higher NOP58 expression correlated with greater drug sensitivity. These findings suggest a broad impact of NOP58 on modulating responses to targeted therapies. Additionally, Figure 9I demonstrates that high NOP58 levels are linked to increased sensitivity to Isoquercitrin, further reinforcing the gene’s role in influencing drug efficacy. These results highlight the potential of NOP58 as a biomarker for predicting therapeutic response to various anticancer agents. This comprehensive drug sensitivity analysis suggests that NOP58 could serve as a critical determinant in optimizing cancer treatments, offering valuable insights into personalized therapeutic strategies based on gene expression profiling.

### Analysis of NOP58 expression and its impact on prostate cancer cell ;lines LNCaP and PC3

**FIGURE 10:**
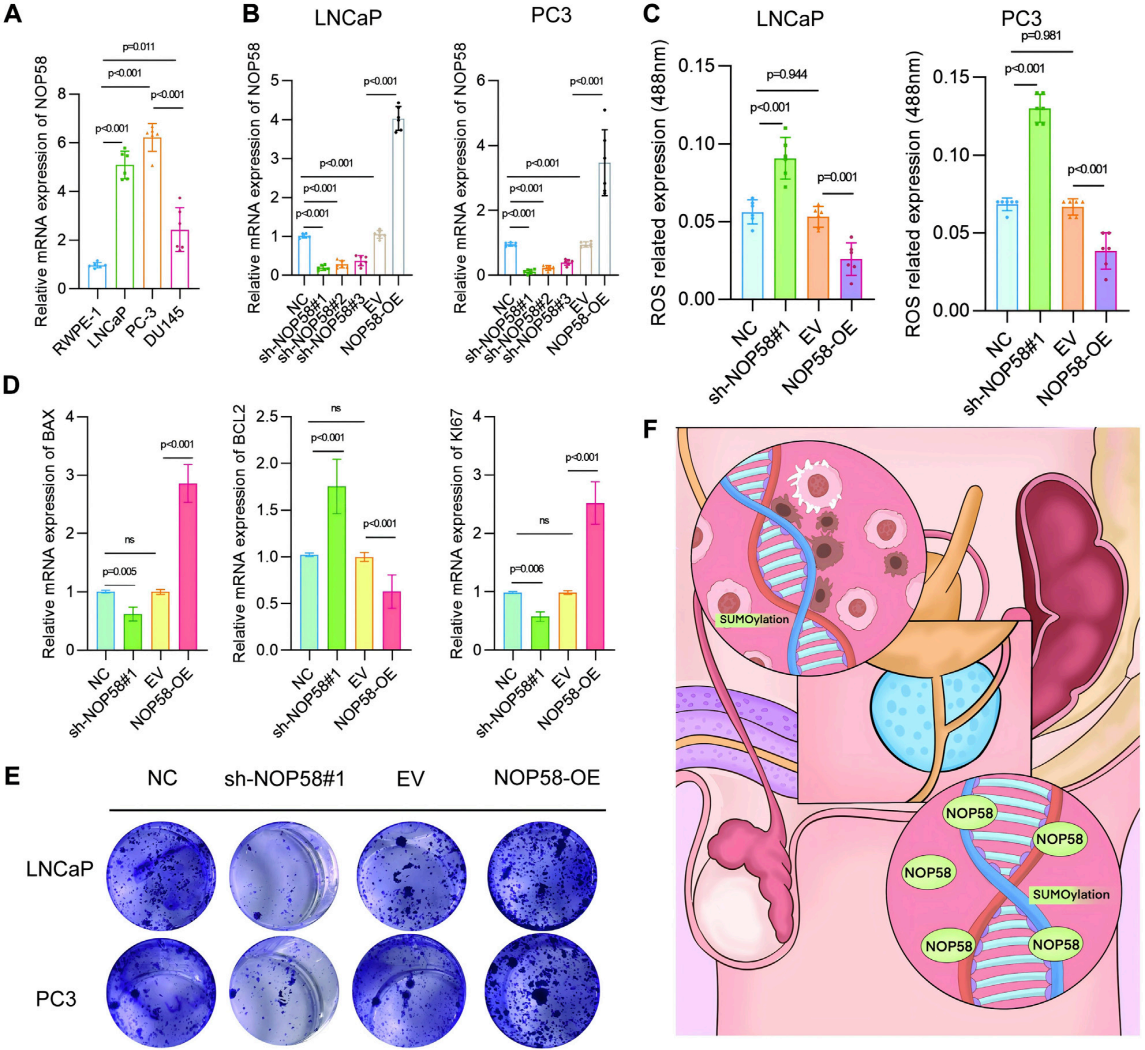
Analysis of NOP58 expression and its impact on prostate cancercCell lines LNCaP and PC3 **(A)** Relative mRNA expression levels of NOP58 in prostate cancer cell lines (RWPE-1, LNCaP, PC3, DU145). Quantitative PCR results are presented as mean ± SD, with statistical significance (p < 0.01). **(B)** NOP58 knockdown and overexpression in LNCaP and PC3 cells. Quantitative PCR results showing significant changes in NOP58 expression following shRNA knockdown (sh-NOP58#1) and overexpression (NOP58-OE) (p < 0.01). **(C)** Flow cytometry analysis of reactive oxygen species (ROS) levels following NOP58 knockdown and overexpression in LNCaP and PC3 cells (**p < 0.01). **(D)** Western blot and quantitative PCR analysis of BCL2 and Ki67 expression in NOP58 knockdown and overexpression cells, showing significant changes (p < 0.01). **(E)** Colony formation assay in LNCaP and PC3 cells showing reduced colony formation following NOP58 knockdown and increased colony formation with NOP58 overexpression.

First, the expression of NOP58 mRNA in various prostate cancer cell lines, including RWPE-1, LNCaP, and PC3, was examined through RT-PCR. Quantitative PCR results indicated that NOP58 was significantly overexpressed in the cancerous LNCaP and PC3 cell lines compared to the noncancerous RWPE-1 line (Figure 10A). To explore the functional role of NOP58, knockdown and overexpression strategies were employed in LNCaP and PC3 cells to modulate NOP58 expression. Quantitative PCR confirmed that NOP58 expression was markedly reduced following knockdown (sh-NOP58#1) and elevated upon overexpression (NOP58-OE). Silencing NOP58 (sh-NOP58#1) resulted in a significant decrease in NOP58 levels (Figure 10B), while overexpression led to a notable increase in its levels (p < 0.01). Subsequently, the effect of NOP58 knockdown on reactive oxygen species (ROS) production in LNCaP and PC3 cells was assessed. Flow cytometry analysis revealed a significant increase in ROS fluorescence following NOP58 knockdown (sh-NOP58#1), while NOP58 overexpression (NOP58-OE) led to a clear reduction in ROS levels (Figure 10C). These findings suggest that NOP58 plays a role in regulating oxidative stress in prostate cancer cells *in vitro*. Further investigations focused on the influence of NOP58 on apoptosis and cell proliferation. BCL2 and Ki67 expression levels were measured, with quantitative PCR confirming that BCL2 levels increased and Ki67 levels decreased after NOP58 knockdown (sh-NOP58#1) (Figure 10D). Conversely, Ki67 levels were significantly upregulated and BCL2 levels downregulated in NOP58-OE cells compared to controls. A colony formation assay was conducted to assess the functional outcomes of NOP58 modulation in LNCaP and PC3 cells. The results showed a substantial reduction in colony numbers following NOP58 knockdown (sh-NOP58#1), while overexpression of NOP58 (NOP58-OE) led to a dramatic increase in colony formation (Figure 10E). In conclusion, these findings underscore the critical role of NOP58 in the proliferation and survival of prostate cancer cells. NOP58 appears to regulate key processes, including oxidative stress response and apoptosis, as demonstrated by its effects on cell-based assays targeting major prostate cancer growth-related pathways. This study highlights NOP58’s involvement in the regulation of oxidative stress, apoptosis, and proliferation in the LNCaP and PC3 prostate cancer cell lines. Dysregulation of NOP58 expression impairs prostate cell function, suggesting that modulating NOP58 levels could be a promising strategy for improving prostate cancer treatment.

## Discussion

The current study aimed to investigate the role of SUMOylation in prostate cancer prognosis and to identify key genes associated with this modification (Sun et al., 2023; Li et al., 2021). Among these genes, NOP58 emerged as particularly significant in prostate cancer progression (Malik and Feng, 2016; Vellky et al., 2021). This conclusion was supported by multiple approaches, including differential expression analysis, survival analysis, GSEA, and single-cell transcriptomics (Špendl et al., 2023; Ma et al., 2020). Survival analysis revealed that overexpression of NOP58 was significantly correlated with poor clinical outcomes, including overall survival (OS), progression-free interval (PFI), and disease-specific survival (DSS) across various cancers. Notably, in these studies, higher expression of NOP58 was associated with worse prognosis (Chen et al., 2024a; Zhang et al., 2024a). Survival analysis revealed that overexpression of NOP58 was significantly correlated with poor clinical outcomes, including overall survival (OS), progression-free interval (PFI), and disease-specific survival (DSS) across various cancers. Notably, in these studies, higher expression of NOP58 was associated with worse prognosis (Xiong et al., 2024; Zhao et al., 2023). Gene ontology and pathway analyses identified crucial biological functions and molecular pathways influenced by NOP58, many of which are closely related to cancer development processes, such as cell cycle progression, DNA repair, and apoptosis (Zhang et al., 2024b; Gao et al., 2024). Results from single-cell RNA sequencing indicated that NOP58 exhibits a high level of heterogeneity across different cellular contexts and interacts with the tumor microenvironment, paving the way for new precision therapy approaches (Li et al., 2022; Wu et al., 2021). These findings align with previous studies suggesting that SUMOylation promotes cancer development (Du et al., 2021; Han et al., 2018). Overall, our study not only provides preliminary evidence of NOP58’s specific role in prostate cancer prognosis but also suggests that NOP58 could be utilized as a diagnostic or therapeutic biomarker for prostate cancer patients (Dimakakos et al., 2014; Adamaki and Zoumpourlis, 2021).

SUMOylation, a key post-translational modification, is indispensable for regulating the activity and degradation of target proteins by attaching small ubiquitin-like modifier (SUMO) proteins (Eifler and Vertegaal, 2015; Raju, 2019). This process affects numerous critical physiological functions, including transcriptional regulation, DNA repair, and signal transductio (Soutourina and Werner, 2014; Puc et al., 2017). This process affects numerous critical physiological functions, including transcriptional regulation, DNA repair, and signal transductio (Chen et al., 2024b). Collectively, the evidence strongly suggests that dysregulated SUMOylation in cancer cells may be a key mechanism driving carcinogenesis and tumor progression (Han et al., 2018; Xie et al., 2020). Through this study, we further elucidated the relationship between SUMOylation and prostate cancer by correlating NOP58 gene expression with clinical outcomes (Sun et al., 2023; Wang and Yu, 2021).

Through this study, we further elucidated the relationship between SUMOylation and prostate cancer by correlating NOP58 gene expression with clinical outcomes (Golomb et al., 2014; Castle et al., 2010). Through this study, we further elucidated the relationship between SUMOylation and prostate cancer by correlating NOP58 gene expression with clinical outcomes (Arriaga-Canon et al., 2018; Ghafouri-Fard et al., 2020). Through this study, we further elucidated the relationship between SUMOylation and prostate cancer by correlating NOP58 gene expression with clinical outcomes (Yu et al., 2021; Nguyen et al., 2023). Given NOP58’s critical role in the SUMOylation pathway, targeting this protein could offer therapeutic efficacy (Kukkula et al., 2021; Kroonen and Vertegaal, 2021). Modulating NOP58 expression or function may interfere with the SUMOylation pathway, thereby inhibiting prostate cancer cell growth and migration (Vlachostergios and Papandreou, 2012; He et al., 2015). This study highlights the novel role of NOP58 as a target of SUMOylation and its regulatory mechanisms in prostate cancer, revealing potential new molecular pathways. In summary, these findings underscore the essential role of NOP58 in prostate cancer and its association with the SUMOylation pathway (Wang and Yu, 2021; Sutinen et al., 2014). As a prognostic marker and therapeutic target, NOP58 provides new directions for prostate cancer research and clinical intervention (Arriaga-Canon et al., 2018; Adamaki and Zoumpourlis, 2021). Future studies will further explore its specific molecular mechanisms and clinical application feasibility, bringing new hope and treatment strategies to prostate cancer patients (Cui et al., 2024).

In this study, we continuously explore and develop new therapeutic strategies by integrating multiple research techniques, including machine learning, multi-omics analysis, three-dimensional reconstruction, and deep learning, providing new possibilities for precision medicine and personalized therapy (Kuo et al., 2024; Sheng et al., 2024). Notably, we combined machine learning algorithms and statistical models to confirm the potential of NOP58 as a prognostic marker for prostate cancer (Kim et al., 2021). High expression of NOP58 is associated with poorer patient prognosis, providing a theoretical foundation for personalized medicine (Wan et al., 2024; Chen et al., 2024c). Studies have shown that the expression of NOP58 in prostate cancer cells and animal models is significantly related to disease aggressiveness and patient survival, validating its value as an independent prognostic predictor (Zhao et al., 2016; Glinsky et al., 2004). The importance of cell death and metabolic regulation in disease progression is increasingly recognized, offering new targets and strategies for NOP58-targeted therapeutic approaches such as small molecule inhibitors or RNA interference technology (Chen et al., 2024d; Zhang et al., 2024c; Zhang et al., 2024d). Although our study has made significant progress, there are some limitations. For instance, while the use of TCGA data is comprehensive, it may not fully represent the genetic diversity of all prostate cancer patients (Li et al., 2014; Cai et al., 2021). Additionally, potential biases in data selection and analysis methods may affect the accuracy of the results (Shringarpure and Xing, 2014; Freed, 2019). Dependence on computational tools and models may introduce potential errors, and the predictive accuracy of NOP58 as a prognostic marker needs to be experimentally validated in larger independent cohorts to confirm our findings (Colită et al., 2024). Therefore, the discovery of NOP58 has significant clinical implications, allowing patient stratification based on risk and guiding personalized treatment strategies (Benson, 2016; Pawlyn and Davies, 2019). Its role in the SUMOylation pathway provides a potential therapeutic target, paving the way for new interventions aimed at regulating this pathway to improve patient prognosis (Huang et al., 2023; Martio et al., 2023; Du et al., 2024).

With the continuous advancement of biomedical research technologies, especially the widespread application of big data and bioinformatics, the accuracy of disease diagnosis and prognosis assessment has been significantly improved (Department of Mechanical and Manufacturing Engineering et al., 2022; Cremin et al., 2022). By integrating clinical and genomic data, researchers have developed various predictive models and tools to forecast disease progression and treatment response (Jiang et al., 2023; Du and Liu, 2024; Li et al., 2024; Yao et al., 2024). These technologies play a core role not only in the identification and application of biomarkers but also in providing critical insights into understanding complex biological processes (McDermott et al., 2013; Dar et al., 2023). For instance, researchers can employ machine learning and deep learning techniques to develop novel predictive models for both short-term postoperative complications and long-term patient prognosis (Cui et al., 2024). The combined use of these advanced techniques not only enhances the depth and breadth of research but also provides a crucial foundation for subsequent clinical applications (Zhang et al., 2023b). Our study contributes to cancer research by integrating multi-omics data with advanced bioinformatics tools (Lu and Zhan, 2018; Lin et al., 2022; Sun et al., 2022a; Sun et al., 2022b). The integrative strategy developed here is not only applicable to prostate cancer but can also be extended to other cancers, offering a comprehensive view of the biological pathways involved in tumorigenesis. This approach has the potential to identify new biomarker candidates and therapeutic targets (Nevedomskaya and Haendler, 2022; Felgueiras et al., 2014). Further studies are required to confirm the value of NOP58 as a prognostic indicator in larger, more diverse patient populations. We also explored the molecular mechanisms of NOP58 and its role in SUMOylation pathways. NOP58 may interact with other molecular pathways, providing further insights into its impact on the tumor microenvironment and prostate cancer biology (McAllister et al., 2019; Corn, 2012). Moreover, NOP58 and its associated signaling cascades could represent promising targets for novel treatment strategies (Qin et al., 2023). Additionally, it has been shown that social support systems positively influence the mental health of cancer patients (Tian et al., 2021a; Tian et al., 2021b). Through comprehensive studies on patient engagement and social support, researchers have demonstrated that these factors significantly contribute to disease management and mental health outcomes (Lamore, 2024; Zhu, 2024). This could be another important consideration in future studies of NOP58 and its broader implications.

## Conclusion

In conclusion, our study highlights the critical role of NOP58 in the progression of prostate cancer and suggests that it may serve as both a prognostic biomarker and a therapeutic target for the treatment of this disease. By integrating multi-omics data with deconvolution and transcription factor-pathway interaction analyses, as well as validating our findings through qPCR, we have provided a comprehensive characterization of the key driver genes underlying prostate cancer using advanced bioinformatics platforms. Our findings contribute to the ongoing efforts to develop personalized medical approaches and treatments for patients, ultimately aiming to improve patient outcomes.

## Data Availability

The original contributions presented in the study are included in the article/Supplementary Material, further inquiries can be directed to the corresponding author.

## Supplementary material

SUPPLEMENTARY FIGURE S1 Cell Type Localization, Gene Expression Differentials, and Immune Therapy Prediction Using NOP58 Expression **(A)** Deconvolution of spatial transcriptomics data, showing the spatial localization of all cell types. Each subplot represents a different cell type, with color gradients indicating the density and distribution of cell types within the tissue. **(B, C)** Differential expression of specific genes in different regions: malignant, mixed malignant, and normal. These plots highlight spatial expression differences of particular genes, with color scales representing expression levels. **(D)** Receiver Operating Characteristic-Area Under the Curve (ROC-AUC) analysis for predicting immune therapy responses based on NOP58 expression. The ROC curve evaluates the ability of NOP58 expression to distinguish between responders and non-responders to immune therapy. **(E, F)** Spearman correlation between NOP58 gene expression and drug sensitivity (IC50 or AUC values) from GDSC1 and GDSC2 databases, showing the potential relationship between NOP58 expression and drug response.
