# Supplementary figures and images for: The role of NOP58 in prostate cancer progression through SUMOylation regulation and drug response
Source: Front Pharmacol. 2024 Oct 18;15:1476025. doi: 10.3389/fphar.2024.1476025 (PMC11530994; doi:10.3389/fphar.2024.1476025)

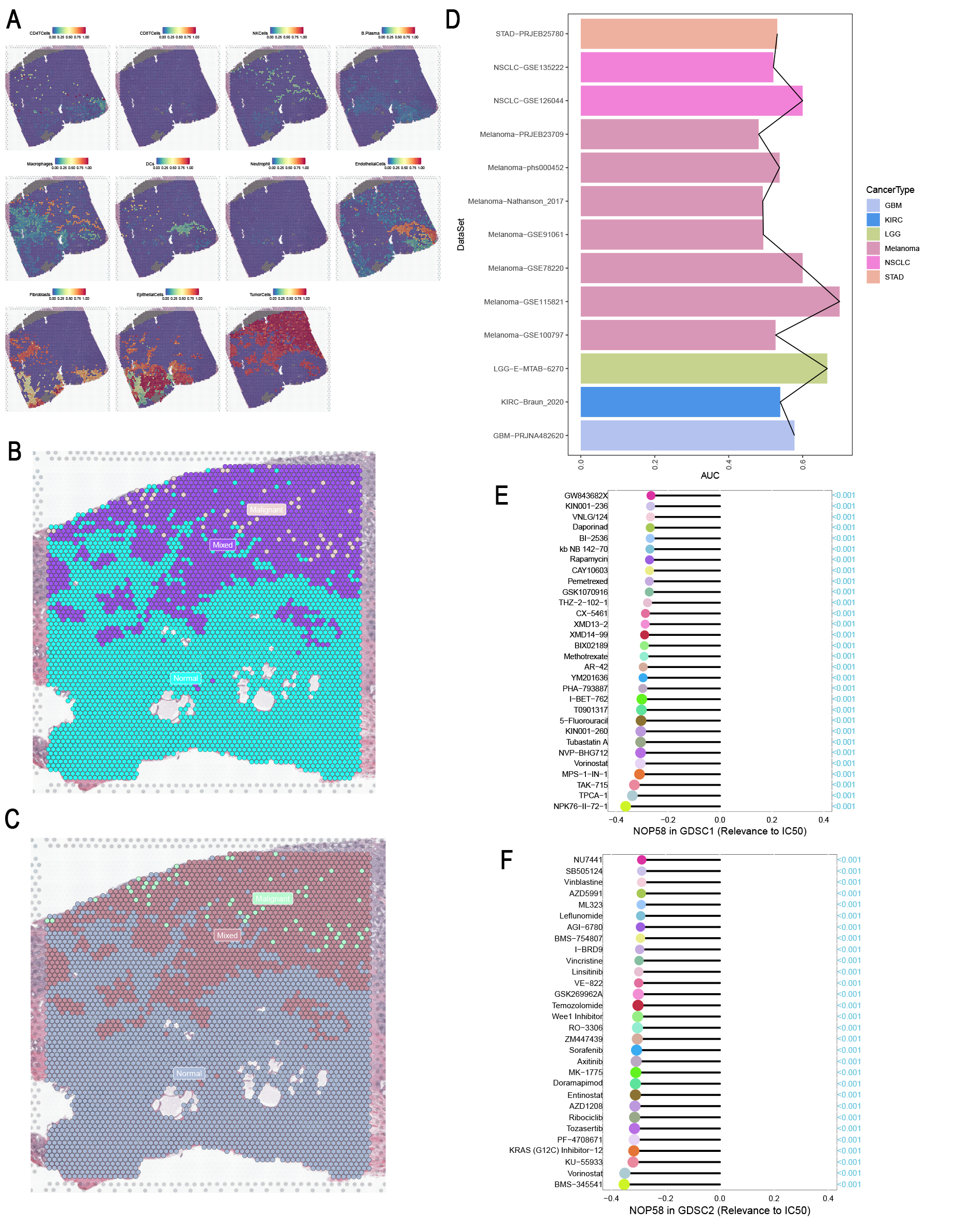

Supplement: Supplementary file 1 [file Image1.PNG]
