# Supplementary material for: The role of NOP58 in prostate cancer progression through SUMOylation regulation and drug response
Source: Front Pharmacol. 2024 Oct 18;15:1476025. doi: 10.3389/fphar.2024.1476025 (PMC11530994; doi:10.3389/fphar.2024.1476025)
Supplement: Supplementary file 2 [file DataSheet1.docx]

**Supplementary methods**

The spatial transcriptomic single-cell data used in this study was sourced from [10x Genomics](https://www.10xgenomics.com/cn/datasets/human-prostate-cancer-adenocarcinoma-with-invasive-carcinoma-ffpe-1-standard-1-3-0). To assess the cellular composition at each point on the 10x Visium slide, we employed deconvolution analysis, integrating spatial transcriptomics (ST) with single-cell RNA sequencing (scRNA-seq) data while considering specific cancer types. We collected scRNA-seq data from multiple samples to construct a comprehensive reference library, applying stringent quality control measures based on gene count, unique molecular identifier (UMI) counts, and mitochondrial RNA proportions. Drawing from relevant studies, we established selection criteria. Subsequently, we constructed a signature score matrix by calculating the average expression levels of cell type-specific genes at each location. Finally, we generated an enrichment score matrix using relevant functions from the Cottrazm package, providing support for the cellular composition analysis.
